# Effectiveness of the ALMA Intervention on Cognitive Function in Women with Breast Cancer: Protocol for a Randomized Controlled Trial

**DOI:** 10.3390/jcm15134876

**Published:** 2026-06-23

**Authors:** Sarah Rebeca Teixeira de Sousa, Juan Luis Sánchez-Rodríguez, Alba Sánchez-Gil, Celia Sánchez-Gómez, Nuria Arroyo-Garrapucho, Emilio Fonseca-Sánchez, Luis Figuero-Pérez, Juan Luis Sánchez-González, Eduardo José Fernández-Rodríguez

**Affiliations:** 1 Programa de Doctorado de Neurociencias, Universidad de Salamanca, Patio de Escuelas, 1, 37008 Salamanca, Spain; sarahsoousa@usal.es; 2 Departamento de Psicología Básica, Psicobiología, Metodología y Ciencias del Comportamiento, Facultad de Psicología, Universidad de Salamanca, Av. de la Merced, 109, 37005 Salamanca, Spain; 3 Centro de Referencia Estatal Centro de Referencia Estatal de Atención de Personas con Enfermedad de Alzheimer y otras Demencias (CREA), C. Cordel de Merinas de Chamberí, 117, 37008 Salamanca, Spain; 4 Departamento de Psicología Evolutiva y de la Educación, Universidad de Salamanca, Instituto de Investigación Biomédica de Salamanca (IBSAL), Campus Miguel de Unamuno s/n, 37007 Salamanca, Spain; celiasng@usal.es; 5 Departamento de Estadística, Universidad de Salamanca, Instituto de Investigación Biomédica de Salamanca (IBSAL), Campus Miguel de Unamuno s/n, 37007 Salamanca, Spain; 6 Departamento de Oncología Médica, Hospital Universitario de Salamanca, Instituto de Investigación Biomédica de Salamanca (IBSAL), 37007 Salamanca, Spain; 7 Departamento de Medicina, Universidad de Salamanca, Instituto de Investigación Biomédica de Salamanca (IBSAL), Campus Miguel de Unamuno s/n, 37007 Salamanca, Spain; 8 Departamento de Enfermería y Fisioterapia, Universidad de Salamanca, Instituto de Investigación Biomédica de Salamanca (IBSAL), Campus Miguel de Unamuno s/n, 37007 Salamanca, Spain; edujfr@usal.es

**Keywords:** breast cancer, cancer-related cognitive impairment, cognitive intervention, randomized controlled trial

## Abstract

**Background**: Cancer-related cognitive impairment is a frequent and clinically relevant concern among women with breast cancer, particularly during active oncological treatment, with potential consequences for memory, attention, executive functioning, daily autonomy, emotional well-being, and quality of life. This study aims to evaluate the effectiveness of the Playful Attention and Active Memory intervention (ALMA) on cognitive functioning in women with breast cancer undergoing active oncological treatment. **Methods**: This single-centre, three-arm, parallel-group randomized controlled trial at the University Healthcare Complex of Salamanca (Spain) will evaluate 63 women with breast cancer undergoing active oncological treatment. Participants will be randomized (1:1:1) into a health education control group, an individual non-tailored cognitive training group, or the ALMA multidimensional group intervention (two 120 min face-to-face sessions/week for four months, combining psychoeducation, targeted cognitive stimulation, and group feedback). Assessments will occur at baseline and post-intervention. The primary outcome is objective global cognitive performance (Montreal Cognitive Assessment). Secondary outcomes include perceived cognitive function, everyday cognition, functional autonomy, anxiety, sleep quality, performance status, and everyday memory failures. Intention-to-treat analysis using linear mixed models will perform prespecified comparisons of ALMA versus both other groups. **Expected results**: This study is designed to provide evidence on the potential value of a structured, multidimensional cognitive intervention delivered during active breast cancer treatment. By comparing ALMA with both health education and individual cognitive training, the trial may clarify whether the integration of psychoeducation, ecological cognitive stimulation, and group-based support offers additional benefits beyond cognitive practice alone. The inclusion of objective, subjective, and functionally oriented outcomes strengthens the clinical relevance of the protocol and may contribute to the development of more comprehensive supportive care strategies for cancer-related cognitive impairment. Trial registration: This protocol is registered at ClinicalTrials.gov under the identifier NCT07165912.

## 1. Introduction

Breast cancer is among the most prevalent malignancies affecting women worldwide, representing a significant public health concern, particularly in Spain [1,2]. It is characterized by the uncontrolled growth of malignant cells in breast tissue, with the potential to invade surrounding structures and metastasize to distant organs [3]. Advancements in early detection and oncological treatment have led to improvements in survival rates, prompting a shift in clinical focus towards the short- and long-term consequences of cancer and its treatment [2,4,5]. Among these consequences, cancer-related cognitive impairment (CRCI), commonly referred to as “chemobrain,” has received increasing attention because of its potential impact on daily functioning, autonomy, emotional well-being, and quality of life [4,5,6,7,8,9].

Cancer-related cognitive impairment may involve difficulties in memory, attention, processing speed, executive functioning, and perceived cognitive efficiency [9,10,11,12,13,14,15]. These difficulties may occur during or after oncological treatment and may persist for months or years in some patients [11,14,15]. Current evidence suggests that CRCI has a multifactorial origin, involving treatment-related neurotoxicity, hormonal changes, inflammatory processes, fatigue, sleep disturbances, anxiety, emotional distress, and other psychosocial factors [12,16,17,18,19,20]. Although the severity and persistence of cognitive symptoms vary across patients, CRCI may interfere with instrumental activities of daily living, social participation, treatment self-management, and return to usual roles [4,19,20].

Non-pharmacological approaches, including cognitive training, cognitive stimulation, psychoeducation, and rehabilitation-oriented interventions, have been proposed as feasible strategies to address CRCI [21,22]. These interventions are designed to enhance cognitive functioning, promote compensatory strategies, and support patients’ perceived control over their daily cognitive challenges [21,22,23]. A recent series of systematic reviews indicates that cognitive rehabilitation and non-pharmacological interventions may improve in terms of cognitive and functional measures in adults diagnosed with cancer. However, the current evidence is characterized by significant heterogeneity in terms of intervention format, intensity, timing, outcome measures, and target population [23,24,25].

An important gap concerns the implementation of structured cognitive interventions during active oncological treatment, a period in which cognitive complaints, fatigue, emotional distress, and treatment-related disruption may be particularly relevant [4,18]. In addition, limited research has directly compared an individual cognitive training programme with a multidimensional group-based intervention combining psychoeducation, cognitive stimulation, and peer interaction. This comparison may help clarify whether the group-based and multimodal components provide additional benefits beyond individual cognitive practice alone [21,24,25].

The present protocol describes a three-arm randomized controlled trial designed to evaluate the Playful Attention and Active Memory intervention (ALMA) in women with breast cancer undergoing active treatment. The ALMA intervention combines psychoeducation, targeted cognitive stimulation, and group feedback, and will be compared with an individual non-tailored cognitive training programme and a health education control condition. The study will assess changes in objective global cognitive performance, perceived cognitive function, everyday cognition, functional autonomy, anxiety, sleep quality, performance status, and everyday memory failures. The inclusion of ecologically valid measures is intended to examine whether potential cognitive changes are reflected in everyday functioning.

The primary objective of this trial is to evaluate the efficacy of the ALMA group intervention in enhancing objective global cognitive performance in women with breast cancer undergoing active oncological treatment, in comparison with individual cognitive training and health education. The primary hypothesis is that participants allocated to the ALMA group intervention (G2) will show greater improvement in MoCA total score from baseline to post-intervention than participants allocated to the health education control group (G0). A second planned hypothesis is that participants allocated to ALMA (G2) will show greater improvement in MoCA total score than participants allocated to the individual cognitive training group (G1), reflecting the potential added value of the multidimensional and group-based components of the ALMA intervention.

## 2. Materials and Methods

### 2.1. Study Design and Setting

This prospective, longitudinal, randomized controlled trial aims to evaluate the efficacy of the ALMA cognitive intervention programme in addressing cancer-related cognitive impairment. The effects of ALMA will be compared with an individual cognitive training programme and a health education control group.

The trial design has been developed in accordance with the Standard Protocol Items: Recommendations for Interventional Trials (SPIRIT) 2013 guidelines [26]. This one-year clinical investigation will be conducted at the University of Salamanca (Spain), specifically within the Department of Medical Oncology at the University Healthcare Complex of Salamanca.

### 2.2. Participants and Eligibility Criteria

Recruitment will be initiated by a qualified oncologist at the University Healthcare Complex of Salamanca, who will identify potential candidates and provide preliminary information regarding the trial. If interested, patients will be scheduled for an in-person consultation with a member of the research team. During this session, the study’s objectives and procedures will be detailed, and any participant question will be addressed.

To determine final eligibility, screening assessment will be conducted. This process will include a baseline research protocol, including a battery of neuropsychological assessments and standardized questionnaires designed to evaluate sleep quality, anxiety symptomatology. Furthermore, written informed consent will be obtained from all subjects prior to any study-related procedures, in accordance with the Declaration of Helsinki.

A summary of the Initial Assessment Protocol and Anamnesis Phase is provided in Table 1. Potentially eligible participants will be identified consecutively and randomized after consent and baseline assessment.

Women aged 18 years and over who have been diagnosed with histologically confirmed breast cancer, at stages I–IV, and who are receiving active oncological treatment at the time of recruitment are eligible to participate. Active treatment will include chemotherapy, radiotherapy, and hormone therapy. Patients with metastatic disease will be included in the study. The time since diagnosis, disease stage, recurrence status, metastatic status, and treatment modality will be recorded as clinical variables rather than used as restrictive eligibility criteria. The specific inclusion and exclusion criteria are described in Table 2 and detailed below:

### 2.3. Recruitment

Participants will be recruited from the Department of Medical Oncology at the University Healthcare Complex of Salamanca. Consecutive identification of potentially eligible women by the oncology team during routine clinical care is to be conducted in accordance with the predefined inclusion and exclusion criteria. Patients who appear to meet the initial eligibility criteria will receive preliminary verbal information about the study. If they express interest, they will be referred to a member of the research team for a detailed explanation of the trial procedures.

The estimated recruitment period will be 12 months, or until the target sample size of 63 participants has been reached. A screening log will be maintained to document the number of patients assessed for eligibility, excluded before randomization, reasons for exclusion when available, number of patients who decline participation, and number of participants randomized to each study arm.

In the event that the intended sample size is not attained within the anticipated recruitment period, the research team may opt to extend the recruitment period, enhance coordination with the oncology service, augment the frequency of screening of outpatient clinic schedules, and provide additional information sessions to eligible patients. Any substantial change in the recruitment strategy, recruitment period, or study setting will be submitted to the Research Ethics Committee for approval when required and will be updated in the trial registry where applicable.

Refusal to participate or withdrawal from the study will not affect participants’ usual oncological care or their relationship with the clinical team.

### 2.4. Sample Size

The planned sample size for this trial is 63 women with breast cancer undergoing active oncological treatment, with 21 participants allocated to each of the three study arms: health education control group (G0), individual cognitive training group (G1), and ALMA group intervention (G2).

The sample size calculation was based on the primary outcome, defined as the change in objective global cognitive performance from baseline (T0) to post-intervention (T1), assessed using the Montreal Cognitive Assessment (MoCA). As no directly comparable previous trial evaluating the ALMA intervention in women with breast cancer was available, the expected effect was estimated using an indirect reference study that evaluated a multicomponent cognitive stimulation programme and reported a mean post-intervention increase of 2.95 points in MoCA score [27]. Assuming a common standard deviation of 3.48, this corresponded to an estimated standardized effect size of Cohen’s d ≈ 0.85.

For the purposes of sample size estimation, this effect was converted to an approximate ANOVA effect size of f ≈ 0.40. The calculation was performed using G*Power software, version 3.1.9.7 [28], for a balanced one-way fixed-effects ANOVA with three independent groups, an alpha level of 0.05, statistical power of 80%, and equal allocation across groups. Under these assumptions, a minimum total sample of 57 participants was required to detect an overall between-group difference.

To account for an anticipated attrition rate of approximately 10%, the recruitment target was increased to 63 participants, corresponding to 21 participants per study arm. This inflation is intended to preserve statistical power for the primary analysis under the intention-to-treat principle.

Although the primary effectiveness analysis will be conducted using linear mixed models to evaluate the Group × Time interaction across baseline and post-intervention assessments, the ANOVA-based sample size calculation was used as a conservative and transparent approximation for planning purposes. Linear mixed models are expected to make efficient use of repeated-measures data and to accommodate missing outcome data under the missing-at-random assumption. Therefore, the ANOVA-based calculation was considered appropriate for estimating the minimum feasible sample size while maintaining consistency with the planned three-arm randomized design.

### 2.5. Randomization and Allocation Concealment

To ensure numerical balance and a homogeneous distribution of prognostic factors across the three study arms, a stratified block randomization method will be employed. Randomization will be stratified based on two baseline prognostic variables: (1) baseline cognitive status, categorized according to the Montreal Cognitive Assessment score (MoCA < 26 vs. ≥26), and (2) oncological treatment modality, classified according to its systemic neurotoxic potential: systemic chemotherapy with or without radiotherapy versus endocrine/hormone therapy or localized radiotherapy alone.

Following stratification, participants will be randomly assigned to one of the three study arms using randomly varied blocks of size 3 and 6. The random allocation sequence will be generated before the start of recruitment by an independent researcher using Epidat 4.2 software. This researcher will not be involved in participant recruitment, eligibility assessment, baseline or post-intervention outcome assessment, intervention delivery, data management, or statistical analysis.

The random allocation sequence will be stored in a password-protected file and will remain inaccessible to the Principal Investigator, oncology team, intervention providers, outcome assessors, and statistician until eligibility has been confirmed, written informed consent has been obtained, and all baseline assessment procedures (T0) have been completed.

After completion of T0, the Principal Investigator will access the next available allocation within the corresponding stratified stratum and assign the participant to the allocated study arm. The assigned group will then be communicated to the intervention coordinator. The allocation will be recorded in a secure allocation log maintained separately from the assessment database.

This procedure will ensure that group allocation cannot influence eligibility confirmation, informed consent, or baseline assessment. Because the Principal Investigator will become aware of group allocation after T0, this trial will be conducted as an open-label study, and this limitation will be addressed through standardized assessment procedures and blinded statistical analysis using neutral group labels.

The three study arms are defined as follows:

- G0 (Control Group): Health Education Programme.
- G1 (Active Comparison): Cognitive Training (CT).
- G2 (Experimental Group): ALMA.

### 2.6. Intervention

#### 2.6.1. Health Education Programme G0 (Control Group)

Participants assigned to the control group (G0) will receive a standardized informational leaflet focused on evidence-based lifestyle modifications. This material will outline comprehensive instructions and recommendations for maintaining an active lifestyle, promoting self-care, and fostering healthy habits.

The content will be aligned with World Health Organization recommendations on risk reduction in cognitive decline and dementia [29]. The key areas covered in the intervention include:

- Nutritional optimization: Adoption of a balanced and nutritious diet.
- Physical & Mental Activity: Regular exercise and engagement in cognitively stimulating tasks.
- Psychosocial Factors: Maintenance of robust social connections and effective stress management techniques.
- Sleep Hygiene: Strategies for ensuring restorative and adequate sleep.
- Metabolic & Toxic Habits: Proper hydration, smoking cessation, and the avoidance of excessive alcohol consumption.

Strictly following the study design, this group will not receive cognitive individual or group stimulation. This group serves as a baseline comparison to evaluate the natural progression of cognitive symptoms under standard care and general health recommendations.

To capture the true, unaltered natural progression of neurocognitive symptoms under standard oncological care, the G0 group follows a passive, no-contact design between evaluations. While this limits real-time behavioral tracking, it deliberately prevents the introduction of reactive biases or artificial behavioral modifications often triggered by frequent researcher contact (the Hawthorne effect). Nonetheless, any spontaneous or clinically significant changes in participants’ lifestyle, sleep hygiene, or psychological status during this period will be captured and quantified via the multi-domain secondary psychometric outcomes (such as the PSQI and Hamilton scales) administered during the post-intervention assessment (T1).

#### 2.6.2. Cognitive Training Group (CT) G1

Participants in the Cognitive Training (CT) group (G1) will undergo a structured programme focused on everyday cognition, administered individually to each patient. The intervention will be delivered through a meticulously developed dossier designed specifically for the objectives of this study. Notably, all participants in this arm will receive identical tasks, with no individualized adaptations for difficulty level or functional profile.

The intervention is structured into four consecutive one-month training periods (P1–P4), totaling 80 activities (20 activities per period). Participants are required to complete five activities per week. To monitor treatment adherence and ensure compliance with therapeutic guidelines, a dual follow-up system will be implemented:

- Remote Follow-up: Conducted via video call at the conclusion of P1 and P3 (Months 1 and 3).
- In-person Follow-up: Conducted at the clinic at the conclusion of P2 and P4 (Months 2 and 4) to evaluate progress and address emergent concerns.

The final evaluation for G1 will coincide with the completion of the fourth training period (P4). This group serves to control the effects of cognitive stimulation and professional monitoring without the specific multimodal components of the ALMA.

#### 2.6.3. ALMA Group Intervention G2

The ALMA intervention is designed as a cognitive intervention programme tailored specifically for breast cancer patients. The primary objective is to address the effects of commonly known chemobrain by targeting cognitive domains commonly affected in CRCI. Beyond cognitive recovery, the programme aims to support self-esteem and patient empowerment through personalized and ecologically valid activities.

The intervention will be delivered through face-to-face group sessions at the Faculty of Psychology of the University of Salamanca. Each ALMA group will include up to six participants. If more than six participants are allocated to G2, additional groups will be created and delivered using the same intervention manual, session structure, materials, and monitoring procedures.

Group size will be limited to six participants to facilitate interaction, individualized support, and consistency in intervention delivery. The programme will span four months, with two 120 min sessions per week, resulting in a total of 32 sessions. The ALMA intervention is characterized by the following components:

- Targeted Cognitive Stimulation: Structured exercises focusing on memory, executive functions, attention, language, and mathematical/logical reasoning with dynamic adjustments to address specific patient deficiencies.
- Neuro-education & Health Literacy: Integrative discussions on neuroscience, cognitive health, and overall well-being to foster self-management.
- Group Dynamics: A collaborative environment designed to facilitate social support and emotional resilience among peers.

The activities will be adaptively tailored to the participants’ functional profiles, ensuring that the stimulation is both accessible and clinically relevant.

To ensure the replicability of the ALMA intervention, the operational components, session structure, materials, and monitoring procedures will be standardized and described in detail in Supplementary Material S1.

Each session will last 120 min and will follow a structured format consisting of welcome and psychoeducation, cognitive intervention, and review and wrap-up. Each session will follow a structured schedule to support consistency:

- Welcome and Psychoeducation (25 min): The session will commence with a “check-in” to assess the well-being of all participants. Subsequently, the group will engage in a discussion of neuropsychological topics with a particular focus on the phenomenon known as “chemobrain.”
- Cognitive Intervention (70 min): Participants will engage in cognitive exercises encompassing the domains of memory, attention, language, executive functions, and mathematical/logical reasoning.
- Review and Wrap-Up (25 min): A thorough explanation and comprehensive review of the completed activities will be provided, along with the group’s feedback.

In terms of the progression of difficulty and the roadmap, the intervention will not be static but will follow a curricular progression.

The exercises will be adapted to the patients’ particular impairments and functional profiles, with the activities structured primarily based on their subjective complaints, prioritizing the domains with the most significant perceived deficits.

The principal investigator will prepare the worksheets containing the cognitive stimulation activities for each session, as well as the psychoeducation topics. The materials to be used in the intervention consist of cognitive stimulation activity worksheets, writing implements, erasers, colored pencils, audio equipment, and the previously referenced games. The implementation of particular materials, including cognitive stimulation activities and games, will help ensure that all participants receive equivalent stimulation, notwithstanding the formation of additional replication groups in the future.

Mechanisms will be established to ensure the consistency and rigor of this study. Consequently, the intervention will adhere to the predetermined clinical framework for Group G2. Furthermore, adherence will be documented through systematic monitoring of attendance using a follow-up log with standardized codes for absences. A success threshold will be applied, requiring a minimum attendance rate of 70% (22 out of 32 sessions) to validate the intervention. Furthermore, a meticulous record of the reasons for withdrawal will be maintained to assess the feasibility of the protocol.

To guarantee the fidelity of the intervention and its replicability in future groups, a manual describing the ALMA Protocol will be created. This manual will be based on a guide developed by the researcher. The guide will outline the objectives, content, and duration of each of the 32 sessions. This manual is designed to ensure that the approach to neuroeducation, cognitive intervention, and group dynamics remains consistent throughout the 4-month period. The progression of the activities will be documented, and the activities will be adapted according to the patients’ functional profiles. This approach is designed to ensure that the stimulation is both effective and primarily focuses on the most affected cognitive functions.

The investigator who is responsible for the interventions will be a professional who has obtained the qualifications necessary for the task.

**Figure 1:**
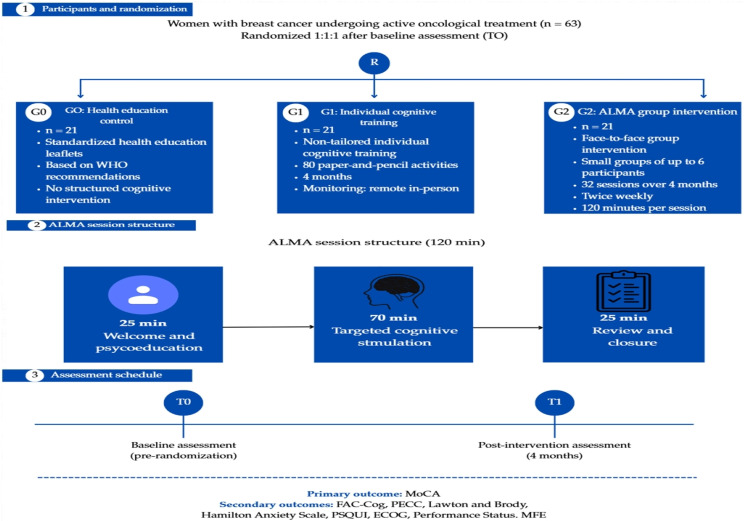
Women with breast cancer undergoing active oncological treatment will be randomized in a 1:1:1 ratio after baseline assessment to health education control (G0), individual cognitive training (G1), or the ALMA group intervention (G2). The ALMA intervention consists of 32 face-to-face sessions delivered twice weekly over four months in small groups of up to six participants. Each 120 min session includes welcome and psychoeducation, targeted cognitive stimulation, and review and closure. Outcomes will be assessed at baseline (T0) and post-intervention (T1). Abbreviations: WHO, World Health Organization; MoCA, Montreal Cognitive Assessment; FACT-Cog, Functional Assessment of Cancer Therapy-Cognition; PECC, Test for the Assessment of Everyday Cognition; PSQI, Pittsburgh Sleep Quality Index; ECOG, Eastern Cooperative Oncology Group; MFE, Everyday Life Memory Failures.

The organizational structure and the specific components of each study arm are summarized in Figure 1. This figure provides a comprehensive overview of the three-group design, highlighting the ALMA protocol as the core clinical framework. It details the internal structure of the G2 sessions comprising psychoeducation, tailored cognitive intervention, and group feedback as well as the longitudinal progression of the four-month intervention roadmap.

### 2.7. Concomitant Care and Co-Intervention

Participants will continue to receive their usual oncological care throughout the study, including chemotherapy, radiotherapy, and hormone therapy. Any relevant changes in oncological treatment during the study period will be recorded as clinical variables. Participation in other structured cognitive rehabilitation or cognitive stimulation programmes during the trial will not be permitted, as it may interfere with the interpretation of intervention effects. Other standard supportive care services will be allowed and documented when relevant.

### 2.8. Participant Enrollment, Withdrawal and Adherence

Adherence to the enrollment and withdrawal guidelines will be checked by systematically recording attendance at all group sessions. A minimum threshold of 70% attendance (22 out of 32 sessions) has been established to consider the intervention complete. To maximize retention and mitigate non-attendance, the following strategies will be implemented:

- Automated Reminders: Notifications will be sent via WhatsApp 24 h prior to each session.
- Scheduling Flexibility: Session hours will be adapted to accommodate participants’ clinical and personal schedules.
- Group Cohesion: The intervention’s group nature is expected to foster social support, reducing the likelihood of dropout.

All absences will be documented in a systematic monitoring log using a binary system (presence/absence) and standardized coding for causes (e.g., clinical discomfort, logistical barriers, or personal reasons). While absences due to medical causes (appointments or post-treatment side effects) will be recorded for qualitative analysis, they will still be included in the final calculation of the 70% adherence threshold.

### 2.9. Withdrawal Criteria

Participants are free to withdraw from the study at any time without providing a justification and without any impact on their standard oncological care. In the event of voluntary withdrawal, the specific causes will be documented to evaluate the intervention’s feasibility.

Following the SPIRIT and CONSORT guidelines, participants who discontinue the intervention will be encouraged to complete the final follow-up assessments to facilitate an Intention-to-Treat (ITT) analysis and minimize bias due to missing data. The Principal Investigator (PI) is responsible for ensuring that all reasons for withdrawal are recorded with precision and anonymity, safeguarding data integrity for subsequent analysis.

### 2.10. Safety Monitoring and Adverse Events

The ALMA and the individual cognitive training programme are considered low-risk non-pharmacological interventions. Nevertheless, potential adverse events may include fatigue, emotional discomfort, frustration during cognitive tasks, increased anxiety related to perceived cognitive difficulties, or temporary distress during psychoeducational discussions about cancer-related cognitive impairment.

Adverse events will be monitored throughout the study period. In G2, the intervention facilitator will ask participants about discomfort or difficulties at the beginning and end of each group session. In G1, potential adverse events will be assessed during remote and in-person follow-up contacts. In G0, adverse events or relevant clinical changes will be recorded at the post-intervention assessment or whenever reported by the participant.

All adverse events will be documented in a standardized log including date, participant code, study arm, description of the event, severity, expectedness, potential relationship with the intervention, action taken, and outcome. Severity will be classified as mild, moderate, or severe. Relatedness will be classified as unrelated, possibly related, probably related, or definitely related to the study procedures. If a participant experiences clinically relevant fatigue, emotional distress, or anxiety during a session, the activity will be paused, and the participant will be offered rest, support, and the option to discontinue the session. If necessary, the participant will be referred to the appropriate oncology or psycho-oncology service according to the usual clinical care pathway.

Serious adverse events, including hospitalization, life-threatening events, or death, will be recorded and reported to the principal investigator and the relevant ethics committee according to institutional requirements. Given the clinical characteristics of the study population, adverse events related to oncological treatment will be distinguished from those potentially related to the study intervention whenever possible.

Participants will be free to withdraw from the study at any time without consequences for their standard oncological care.

### 2.11. Administrative and Oversight Information

This manuscript describes version 1.0 of the ALMA trial protocol, dated June 2026. The trial is registered at ClinicalTrials.gov under the identifier NCT07165912. The trial registry record will be kept consistent with the final approved protocol, including the study design, target sample size, intervention arms, eligibility criteria, outcome measures, and assessment time points.

The Principal Investigator will be responsible for the overall coordination of the trial, protocol implementation, supervision of recruitment, safety reporting, data integrity, and communication with the Research Ethics Committee. The oncology team will identify potentially eligible participants during routine clinical care. The principal researcher will obtain informed consent, administer baseline and post-intervention assessments, maintain study documentation, and monitor adherence. The principal researcher will be responsible for the intervention and deliver the ALMA sessions according to the intervention manual and will document attendance, adverse events, and protocol deviations.

There is no external or commercial sponsor for this study. The University of Salamanca will provide institutional oversight in accordance with applicable ethical, legal, and institutional requirements.

Given the low-risk, non-pharmacological nature of the ALMA intervention and the individual cognitive training programme, no independent Data Monitoring Committee will be established. The Principal Investigator and authorized members of the research team will conduct trial monitoring internally. Monitoring activities will include periodic review of recruitment progress, eligibility documentation, informed consent forms, adherence records, intervention attendance logs, adverse event reports, protocol deviations, withdrawal reasons, and completeness of outcome assessments.

No routine external auditing is planned. However, essential study documents may be reviewed by the University of Salamanca, the Research Ethics Committee, or other competent institutional authorities if required. Documents available for review may include the approved protocol and amendments, ethics approval documentation, informed consent forms, screening and enrolment logs, randomization and allocation records, adverse event logs, protocol deviation records, data management logs, and pseudonymized datasets.

This study is funded by the Salamanca Biomedical Research Institute (IBSAL), Spain, as part of the project “Clinical research in cancer patients”, CODE: L1SN. The funder will have no role in the design of the study, data collection, data analysis, interpretation of results, dissemination, or publication of the trial findings. No commercial funding has been received for this study.

### 2.12. Intervention Materials

**Figure 2:**
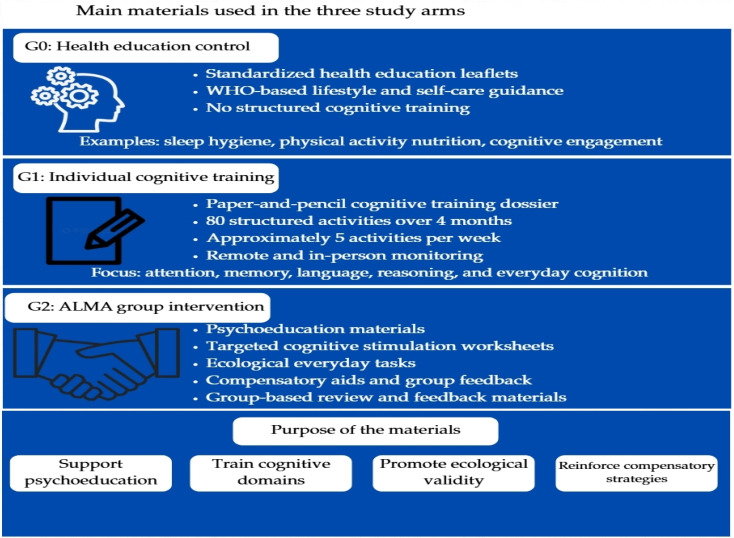
Intervention materials and delivery components across study arms. The figure summarizes the main materials used in each study arm. G0 participants receive standardized health education leaflets based on WHO recommendations. G1 participants complete an individual, non-tailored paper-and-pencil cognitive training dossier. G2 participants receive the ALMA group intervention, which integrates psychoeducation materials, targeted cognitive stimulation worksheets, ecological everyday tasks, compensatory aids, and group-based review and feedback materials. Abbreviations: WHO, World Health Organization; ALMA, Playful Attention and Active Memory.

The intervention materials were designed to reflect the specific objectives of each study arm and to support consistency in intervention delivery. Figure 2 provides an overview of the materials used in G0, G1, and G2, highlighting the psychoeducational, cognitive, ecological, compensatory, and group-based components of the ALMA intervention.

- G0 (Health Education): Participants will receive informational support through standardized leaflets. These materials are based on WHO guidelines and provide evidence-based recommendations on healthy lifestyles, self-care, and risk-reduction practices to mitigate cognitive decline.
- G1 (Cognitive Training): This group utilizes individual dossiers focusing on tasks that simulate Instrumental Activities of Daily Living (IADL). These activities require the integration of multiple cognitive functions, including attention, reasoning, working memory, planning, and processing speed. Specific functional contexts include medication adherence, meal preparation, financial management, and the use of communication technologies.
- G2 (ALMA): The experimental group employs a specialized cognitive intervention framework designed to target all major cognitive domains. This intervention requires a diverse array of multisensory and multimodal materials, including audiovisual support, specialized notebooks, and various stationery (e.g., colored pencils, scissors, and photos).

The use of these materials is intended to support ecological validity by linking cognitive exercises to everyday activities relevant to women with breast cancer.

## 3. Outcome Measures

The effects of the ALMA compared to Cognitive Training (G1) and Health Education (G0) will be evaluated through a comprehensive battery of validated instruments. All assessment tools have been used in clinical or oncological research and validated Spanish versions will be used where available.

Evaluations will be conducted at baseline (T0) and upon completion of the four-month intervention (T1). In order to facilitate the interpretation of the study’s outcomes, Table 3 presents the primary and secondary outcome measures, the instruments that will be used for assessment, the evaluation time points, the scoring direction of each scale, and the direction of change considered to indicate improvement from baseline (T0) to post-intervention (T1).

### 3.1. Outcome Definitions and Scoring Direction

All outcomes will be assessed at baseline (T0) and after the conclusion of the four-month intervention period (T1). For each outcome, change scores will be calculated as the post-intervention score minus the baseline score (T1 − T0). The direction of clinical interpretation depends on the scoring system of each instrument.

For the primary outcome, a positive change in the Montreal Cognitive Assessment (MoCA) total score will indicate improvement in objective global cognitive performance.

While the MoCA is a screening instrument, its designation as the primary endpoint is clinically justified by its widespread validation, brief administration time (minimizing evaluation fatigue in active cancer patients), and extensive use as a benchmark across CRCI trials, allowing for direct cross-study comparisons. Furthermore, the MoCA exhibits superior sensitivity to the specific neurocognitive phenotypes of “chemobrain” compared to other global screeners, as it incorporates rigorous subtests dedicated to executive functions, working memory, sustained attention, and delayed verbal recall. To actively mitigate the inherent risk of Type II errors and address potential ceiling effects in this relatively young and functional patient population, the MoCA is structurally supplemented within this multi-method protocol by robust secondary measures capable of capturing subtle cognitive fluctuations.

For FACT-Cog, higher scores indicate enhanced cognitive functioning and elevated cognitive-related quality of life. For the PECC and the Lawton and Brody Scale, higher scores are indicative of better every day cognitive performance and greater functional autonomy, respectively. Conversely, higher scores on the Hamilton Anxiety Scale, Pittsburgh Sleep Quality Index, ECOG Performance Status Scale, and Everyday Life Memory Failures Questionnaire will indicate worse anxiety symptoms, poorer sleep quality, poorer functional performance status, and more frequent everyday memory failures, respectively.

The primary endpoint will be the between-group difference in change in MoCA total score from T0 to T1. The primary comparison will be between the ALMA group intervention (G2) and the health education control group (G0). Secondary comparisons will include G2 versus G1. The G1 versus G0 comparison, if reported, will be interpreted as exploratory.

### 3.2. Primary Outcome: Objective Global Cognitive Performance

The primary endpoint will be the between-group difference in change in MoCA total score from baseline (T0) to post-intervention assessment (T1), calculated as ΔMoCA = MoCA score at T1 − MoCA score at T0. The MoCA is a brief standardized cognitive screening instrument that evaluates multiple cognitive domains, including executive functions, attention, abstraction, memory, calculation, language, visuospatial abilities, and orientation. The total score ranges from 0 to 30, with an administration time of approximately 10–12 min. To correct for socio demographic educational bias, an additional point is added to the total score for participants with ≤12 years of formal education. The Spanish version exhibits robust psychometric validity, with a reported internal consistency of Cronbach’s α = 0.84 and a test–retest reliability of r = 0.92. A score below the clinical cut-off of <26 points indicates objective cognitive impairment. For cancer-related cognitive impairment (CRCI) monitoring, the Minimal Detectable Change (MDC) is established at ±2 points; thus, an intra-subject post-intervention increase of ≥2 points indicates a true, statistically stable cognitive improvement.

In this study, the MoCA will be used as an objective global measure of cognitive functioning rather than as a diagnostic tool for cancer-related cognitive impairment [30].

Although CRCI may involve subtle and domain-specific cognitive changes, the MoCA was selected as the primary outcome because it provides a standardized, feasible, and clinically interpretable measure of global cognitive performance. To complement this global measure, secondary outcomes will include patient-reported cognitive functioning, everyday cognition, functional autonomy, sleep quality, anxiety, performance status, and everyday memory failures.

### 3.3. Secondary Outcomes

Secondary outcomes will include subjective cognitive function, everyday cognition, functional autonomy, anxiety, sleep quality, performance status, and everyday memory failures.

- Functional Assessment of Cancer Therapy-Cognition (FACT-Cog), Version 3: To assess patient’s perceived cognitive functioning and its impact on quality of life. The FACT-Cog assessment is composed of 37 item questionnaires, which are divided into six cognitive domains: memory, concentration, mental acuity, verbal fluency, functional interference, and multitasking ability. The scale is composed of three subscales: symptoms of perceived cognitive impairment, where a higher score indicates fewer symptoms; perceived cognitive abilities, where a higher score indicates superior cognitive skills rating; and overall quality of life, where a higher score indicates an enhanced quality of life relative to cognition [31]. Validated in Spanish oncology cohorts, it demonstrates exceptional internal consistency α = 0.93 for the global scale α = 0.90 for the CogPCI subscale) and a test–retest reliability coefficient of r = 0.84. Higher total or subscale scores indicate fewer subjective cognitive complaints and a higher cognitive-related quality of life. The MCID for the total score ranges between 6.9 and 10.6 points (and 4.6 points specifically for the CogPCI subscale) to establish a clinically meaningful therapeutic improvement.
- The Test for the Assessment of Everyday Cognition (PECC) is a standardized instrument designed to evaluate an individual’s capacity to manage day-to-day activities. The PECC assesses an individual’s ability to solve 12 real-life scenarios, which are grouped into the following domains: medication, administrative management, financial management, food preparation, transportation, and shopping. The PECC provides a comprehensive assessment of an individual’s functional capacity in daily living activities, thereby providing valuable insights into their overall cognitive abilities and daily living skills. The administration time is 35 min [32]. Normatively validated in Spain for adult populations, the PECC demonstrates an excellent inter-rater reliability with an Intraclass Correlation Coefficient (ICC) of 0.94, alongside robust ecological validity. Scores are calculated continuously per domain and then aggregated, where higher cumulative scores denote superior objective functional autonomy. A statistically significant post-intervention increase relative to the control group demonstrates successful clinical transfer of trained cognitive strategies to the patient’s actual environment.
- The Lawton and Brody Scale is a tool designed to evaluate autonomy in instrumental activities of daily living. The administration time for this assessment is four minutes. It measures 8 semi-complex tasks: telephone use, shopping, food preparation, housekeeping, laundry, mode of transportation, responsibility for own medications, and financial management. Each item is scored binary as either 0 (dependent) or 1 (independent), yielding a total score from 0 to 8 in women [33]. It exhibits high cross-cultural reproducibility with a Guttman scalogram coefficient of 0.96. A total score of 8 indicates complete functional independence. Any score <8 establishes functional decline. Therapeutic success is operationalized as the preservation of maximum baseline autonomy (score of 8) or a reduction of dependency indicators post-chemotherapy.
- The Hamilton Anxiety Scale is a psychometric instrument that is employed to evaluate the severity of anxiety in patients who meet criteria for anxiety [34]. The scale consists of 14 items, each with five response options ranging from “not present” to “very severe” with a total score range of 0 to 56. The adapted Spanish version demonstrates high internal consistency (α = 0.89) and inter-rater reliability (r = 0.94). Clinical severity thresholds are categorized as: ≤17 (mild anxiety), 18–24 (mild-to-moderate anxiety), and 25–30 (moderate-to-severe anxiety). The MDC for this instrument is 4 points; thus, a post-intervention decrease of ≥4 points confirms a statistically reliable and clinically significant therapeutic reduction in anxiety.
- The Pittsburgh Sleep Quality Index (PSQI) was developed to assess sleep quality in individuals between the ages of 24 and 83. This instrument requires approximately 5 to 10 min for completion. The study’s objective is to assess the impact of subjective sleep quality, sleep latency, sleep duration, habitual sleep efficiency, sleep disturbances, sleep medication use, and daytime dysfunction on a total of 19 individual items [35]. Validated in Spanish oncology settings, it displays an internal consistency of α= 0.81 and high diagnostic sensitivity (89.6%). A global score >5 serves as the clinical cut-off point, separating “good sleepers” from “poor sleepers” (chronic sleep pathology). The MDC is 2 points, meaning a downward shift of ≥2 points post-intervention establishes a clinically relevant improvement in sleep hygiene.
- The Performance Status Scale (ECOG): Is a standardized scale used to evaluate the functional status of cancer patients and assess their ability to perform activities of daily living. It allows for an assessment of the disease’s impact on the patient’s functional status and helps guide therapeutic and prognostic decisions. The scale classifies functional status on a scale of 0 to 5. The numeral 0 indicates Patient is fully active, with no restrictions on performing daily activities;1: Limited ability to perform physically strenuous activities, but capable of light or sedentary work; 2: Capable of self-care, though unable to perform work activities; remains active more than 50% of the day; 3: Limited capacity for self-care; remains in bed or a chair more than 50% of the day; 4: Completely disabled; totally dependent for self-care; 5: Death. This scale ensures tight control over confounding physical decline during the trial; maintaining a Grade 0 or 1 throughout the study indicates clinical stability. [36].
- The Everyday Life Memory Failures (MFE) Questionnaire is a tool designed to assess various forms of memory impairment, a 28 item self-report instrument designed to evaluate the subjective frequency of cognitive lapses and minor memory failures occurring within the patient’s typical daily environment. Respondents rate each item on a 5-point Likert scale (0 = never to 4 = very often), with total scores ranging from 0 to 112. It encompasses a range of categories, including verbal memory, reading aptitude, writing skills, recognition of names, faces, and actions, as well as the ability to learn new information. The response to this question is measured using a Likert scale, which provides nine possible responses ranging from “Not once in the last 3 months” to “More than 1 time a day” [37]. The Spanish version exhibits a robust internal consistency (α= 0.89) and a stable test–retest reliability (r = 0.83). Higher cumulative scores reflect a higher density of daily cognitive failures, with normative healthy adult data averaging scores of 20–25. Consequently, a downward change score (Δ MFE < 0) post-intervention demonstrates an increase in perceived daily cognitive control and efficiency.

### 3.4. Clinical and Sociodemographic Variables

In addition to the outcomes above, the analysis will incorporate:

Sociodemographic data: Age, level of education, and marital status.

Clinical profile: Type of oncological treatment (chemotherapy, radiotherapy, hormone therapy), time since diagnosis, and general health status.

### 3.5. Blinding and Allocation Concealment

Due to the nature of the ALMA cognitive intervention, complete blinding of the participants and the therapists delivering the sessions is not possible. Furthermore, assigning an independent assessor for the post-intervention evaluations (T1) is unfeasible due to human resource constraints within the specialized clinical setting and the strict methodological requirement to minimize inter-rater variability during manual neuropsychological testing. To mitigate potential detection bias and safeguard internal validity, several countermeasures have been adopted. First, all primary and secondary outcome measures including the Montreal Cognitive Assessment (MoCA) and the Test for the Assessment of Everyday Cognition (PECC) utilize standardized, highly structured, and objective quantitative scoring systems that eliminate subjective interpretation. Second, manual tests will be supplemented by objective, computerized automated scoring software where applicable to ensure absolute data consistency. Finally, the statistical analysis will be performed by an external biostatistician who will remain completely blinded to group allocation, working exclusively with anonymized datasets.

### 3.6. Participant Evaluation

The evaluation of participants will occur at two distinct time points:

Baseline Evaluation (T0): Conducted immediately after recruitment and prior to randomization. This stage ensures the documentation of all sociodemographic and clinical variables, alongside the administration of objective cognitive tests, without the influence of group assignment.

Post-Intervention Evaluation (T1): Scheduled upon the culmination of the four-month intervention period for all groups.

Following the baseline evaluation, participants will undergo randomization to their assigned intervention arm (ALMA, Cognitive Training, or Health Education).

For a comprehensive overview of the pertinent data, please refer to the table provided in Supplementary Material S2. Table follows the SPIRIT participant timeline structure and reflects the protocol schedule described in the manuscript.

### 3.7. Data Management and Confidentiality

#### 3.7.1. Data Collection, Storage and Confidentiality

To ensure the protection of personal data and maintain participant privacy, a pseudonymization process will be implemented. Upon enrolment, each participant will be assigned a unique alphanumeric study code, which will be used for all research-related documentation, assessment forms, intervention records, and electronic databases. Directly identifiable information will not be included in the analysis dataset.

Data will be collected and managed using REDCap or an equivalent secure institutional electronic data capture platform. Access to the database will be restricted to authorized members of the research team through individual user credentials. The file linking participant identities with study codes will be stored separately from the main research database in an encrypted file accessible only to the Principal Investigator.

Study data will be stored on secure institutional servers or approved institutional platforms with restricted access. All procedures will comply with Regulation (EU) 2016/679, the General Data Protection Regulation, and Spanish Organic Law 3/2018 on the Protection of Personal Data and Guarantee of Digital Rights. No directly identifiable participant information will be included in publications, presentations, reports, or shared datasets.

#### 3.7.2. Data Quality Control, Backup, and Change Management

Data quality will be monitored throughout the study by the Principal Investigator and authorized members of the research team. The electronic database will include predefined variable labels, coding rules, range checks, and validation procedures whenever possible to reduce data entry errors.

The database will be reviewed periodically to identify missing values, out-of-range responses, inconsistent entries, duplicate records, and discrepancies between source documents and electronic records. When inconsistencies are detected, they will be checked against the original study documentation and corrected only by authorized personnel.

Any modification made after initial data entry will be documented, including the date of the change, the person responsible, the original value, the corrected value, and the reason for the modification, where technically feasible. These procedures will allow traceability of changes and support data integrity.

Regular backup procedures will be implemented according to institutional data security policies. Before statistical analysis, the dataset will be reviewed for completeness, internal consistency, and plausibility. A final pseudonymized dataset will be created for analysis, with direct identifiers removed. Any data cleaning decisions, exclusions, or transformations will be documented in a data management log to ensure transparency and reproducibility.

### 3.8. Intervention Fidelity and Reproducibility Protocols

To ensure clinical reproducibility and strict intervention fidelity across current and future cohorts, the dynamic adaptability of the ALMA intervention follows a rigorous, manualized framework of standardized flexibility. While tasks are adaptively calibrated to address patients’ subjective complaints, these modifications are strictly constrained by a pre-established algorithmic matrix that governs task complexity (e.g., pre-defined adjustments in stimulus exposure time, working memory load, or cognitive interference layers). The structural architecture of the sessions including duration, multi-domain cognitive goals, and metacognitive training modules remains strictly invariant across all participants. Intervention fidelity will be systematically monitored using a session-by-session compliance checklist that tracks adherence to manualized timelines, objective delivery, and applied difficulty parameters.

### 3.9. Statistical Analysis

All analyses will be conducted according to the intention-to-treat (ITT) principle, including all randomized participants in the groups to which they were originally allocated, regardless of adherence to the intervention, thereby ensuring that the primary effectiveness analysis remains fully powered even if a significant proportion of participants fail to meet compliance thresholds. A secondary per-protocol analysis may be conducted including participants who complete the post-intervention assessment and, in the intervention arms, meet the predefined adherence criteria (≥70% attendance). In the event that a large majority of participants fail to reach this updated 70% attendance threshold due to severe treatment-related toxicity, a secondary sensitivity dose–response analysis will be implemented, modeling session attendance as a continuous covariate to evaluate the threshold-independent impact of intervention volume on neurocognitive outcomes.

Descriptive statistics will be used to summarize baseline sociodemographic and clinical characteristics by study arm. Continuous variables will be presented as means and standard deviations or medians and interquartile ranges, depending on their distribution. Categorical variables will be presented as frequencies and percentages. Baseline comparability between groups will be described, but statistical testing of baseline differences will not be used to determine participant inclusion or exclusion from the analysis.

The primary effectiveness analysis will evaluate change in Montreal Cognitive Assessment (MoCA) total score from baseline (T0) to post-intervention assessment (T1). Linear mixed models (LMM) will be constructed using a Restricted Maximum Likelihood (REML) estimation approach. The model will include fixed effects for group, time, and the Group × Time interaction term, and a parsimonious random-effects structure restricted to a random intercept for each participant to account for within-subject correlation across time while excluding random slopes. The Group × Time interaction will be the main parameter of interest. Both unadjusted and adjusted models will be calculated to verify the robustness of the intervention effect.

The planned between-group comparisons for the primary outcome will focus on the ALMA group intervention (G2). The first planned comparison will evaluate the difference in change in MoCA total score from T0 to T1 between G2 and the health education control group (G0). The second planned comparison will evaluate the difference in change in MoCA total score from T0 to T1 between G2 and the individual cognitive training group (G1), in order to assess the potential added value of the multidimensional group-based ALMA intervention over individual cognitive training. The comparison between G1 and G0 will not be considered a planned efficacy comparison and, if reported, will be interpreted as exploratory.

To strictly prevent statistical overfitting and safeguard the degrees of freedom given the target sample size of 21 participants per study arm, the adjusted model will be parsimoniously restricted. Specifically, a maximum of two pre-specified baseline covariates will be simultaneously controlled for as fixed effects: chronological age (in years) and educational level (in years of formal schooling). Baseline cognitive stratification variables will be excluded from the model as covariates, given that their equilibrium across groups is already mathematically guaranteed by the stratified block randomization procedure (Section 2.5). Additional clinical and psychological variables including disease stage, treatment modality, time since diagnosis, cancer-related fatigue/performance status, baseline anxiety symptoms (Hamilton Anxiety Scale), and baseline ECOG Performance Status will be exclusively examined in exploratory or sensitivity analyses rather than included simultaneously in the primary adjusted model.

Because two planned comparisons will be conducted for the primary outcome, namely G2 versus G0 and G2 versus G1, Holm–Bonferroni correction will be applied to these two comparisons to control the family-wise error rate. Holm–Bonferroni correction will also be applied to secondary outcome comparisons within each outcome family when appropriate. Any additional post hoc analyses, including G1 versus G0 comparisons, will be clearly labelled as exploratory.

Secondary outcomes will be analyzed using the same modelling framework, with each outcome assessed separately. These outcomes will include perceived cognitive function, everyday cognition, functional autonomy, anxiety symptoms, sleep quality, performance status, and everyday memory failures. For each secondary outcome, the Group × Time interaction will be used to estimate whether change from T0 to T1 differs between study arms. The planned secondary comparisons will focus on G2 versus G0 and G2 versus G1. To account for potential variations in participant insight, changes in subjective memory failures will be systematically triangulated and interpreted alongside the objective primary cognitive trajectories (MoCA scores).

Missing outcome data will be examined descriptively. The primary linear mixed model analysis will assume that data are missing at random. Patterns of missing data will be explored by comparing participants with complete and incomplete outcome data. Sensitivity analyses will include complete-case analyses and, if attrition exceeds 15%, multiple imputation by chained equations. Model assumptions will be assessed by inspecting residual distributions and model diagnostics.

All statistical tests will be two-tailed. Statistical significance for the primary analysis will be set at *p* < 0.05 after Holm–Bonferroni correction for the two planned primary comparisons. Effect estimates will be reported with 95% confidence intervals. Analyses will be conducted using IBM SPSS Statistics version 28.0.1 or equivalent statistical software.

## 4. Discussion

The increasing survival rates in breast cancer have led to a shift in the clinical focus toward long-term treatment side effects, particularly in CRCI. Preliminary research suggests a percentage ranging up to 75% of patients undergoing cognitive alterations during treatment [4,5,9,17,19], which underscores the pressing need for validated, accessible, and early-stage stimulation programmes.

The objective of this research will be twofold: first, to evaluate the efficacy of the ALMA group (G2) and second, to assess the most effective modality for different patient profiles through G0, G1, and G2. In contrast to the other two study arms (G0 and G1), the ALMA group will implement an intervention that includes psychoeducation, direct training on cognitive functions, the identification of internal and external strategies for coping with CRCI, and interaction between the researcher and participants in a structured and stimulating environment.

Furthermore, the implementation of the ALMA intervention during the patients’ active treatment phase will allow for the adaptation of cognitive activities and exercises based on the participants’ subjective complaints, resulting in more structured activities that focus primarily on training the cognitive abilities showing the most deficits.

Based on previous studies and reviews on cognitive rehabilitation, cognitive training, and non-pharmacological interventions for cancer-related cognitive impairment [23,24,38,39,40,41,42], the ALMA intervention is expected to support cognitive, emotional, and functional outcomes. However, the extent to which these potential effects are attributable to cognitive stimulation, psychoeducation, ecological task practice, or group-based support will need to be examined through the planned between-group comparisons.

A strength of this protocol is its ecological validity. In this protocol, ecological validity refers to the extent to which the assessment tools and intervention activities reflect cognitive demands encountered in everyday life. For this reason, the study includes the Test for the Assessment of Everyday Cognition (PECC), which evaluates performance in real-life scenarios such as medication management, administrative tasks, financial management, meal preparation, transportation, and shopping. This measure complements the MoCA by assessing whether potential cognitive improvements are reflected in functional situations that are meaningful for patients. The Lawton and Brody Scale and the Everyday Life Memory Failures Questionnaire further support this ecological approach by assessing instrumental activities of daily living and subjective memory failures in daily life, respectively. In addition, the incorporation of the FACT-Cog ensures that patients’ subjective experience of perceived cognitive difficulties is included in the evaluation of intervention effects.

Furthermore, this study employs a holistic and multifactorial perspective by analyzing anxiety and sleep quality as potential mediators. As documented in the extant literature, emotional distress and poor sleep have been shown to exacerbate symptoms of “chemobrain” by taxing attentional resources and executive functions [14,17,19]. The protocol’s objective is to provide further understanding of individual differences in CRCI by controlling these variables.

### 4.1. Methodological Justification for the Difference Between Groups G0 and G2

The Role of Social Support as a Potential Non-specific Intervention Component: The difference in therapeutic contact between G0 and G2 represents an important methodological consideration. G0 receives informational support only, whereas G2 involves regular face-to-face group sessions, peer interaction, structured professional support, psychoeducation, and cognitive stimulation. Therefore, any positive outcomes observed in G2 should not be interpreted as the effect of cognitive stimulation alone.

Group-based interventions may provide social support and may contribute to emotional regulation, perceived coping, engagement, and cognitive functioning in patients with cancer [23,24,38,39,40,41,42]. In this context, the ALMA intervention should be understood as a multidimensional intervention in which cognitive, psychoeducational, ecological, and group-based components may interact. The comparison between G2 and G0 will therefore estimate the overall effect of the ALMA intervention package compared with health education, rather than isolating the effect of any single component.

Professional Attention and Expectations: The difference in the intensity of professional contact between G0 and G2 may also influence participant engagement, expectations, and perceived support. G2 participants will receive two 120 min face-to-face sessions per week, whereas G0 participants will receive standardized health education materials. This difference reflects the real-world nature of the ALMA intervention but may also introduce non-specific effects related to attention, interaction, and therapeutic contact. For this reason, the comparison between G2 and G1 will be particularly relevant, as G1 includes structured cognitive activities and monitoring but does not include the same group-based and multimodal format as ALMA.

Mitigation Strategy and Design Justification: To address these potential sources of bias, the statistical analysis will include prespecified covariates such as age, education level, and baseline MoCA stratum, with additional clinical and psychological variables examined in exploratory or sensitivity analyses. The planned comparison between G2 and G1 will help assess whether the multidimensional and group-based components of ALMA provide added value beyond individual cognitive training. However, because the trial is not designed to isolate each intervention component separately, findings will be interpreted as reflecting the comparative effectiveness of the intervention packages rather than the independent effect of a single component.

Ecological Validity and Experimental Control: The design prioritizes ecological validity by evaluating ALMA as a complex intervention delivered in a realistic oncology-related context. This approach may reduce experimental purity because the intervention combines cognitive stimulation, psychoeducation, professional support, peer interaction, and ecological task practice. Nevertheless, this design is appropriate for evaluating the feasibility and potential clinical relevance of ALMA as an intervention package for women with breast cancer undergoing active treatment.

### 4.2. G1 and G2

The comparison between G1 and G2 is intended to examine whether the multidimensional and group-based ALMA format provides additional value beyond individual cognitive training. Both groups include cognitive activity, but they differ in delivery format, intensity of therapeutic contact, group interaction, and integration of psychoeducational and ecological components.

G1 is based on the principle that structured and repeated cognitive practice may support specific cognitive domains such as attention, memory, reasoning, and executive functioning. Individual cognitive training activities, including paper-and-pencil exercises, represent a feasible approach to cognitive rehabilitation in cancer survivors [21,23,39,41]. This format allows participants to work individually and at their own pace, without the influence of group dynamics.

By contrast, G2 addresses CRCI from a multidimensional perspective by combining psychoeducation, cognitive stimulation, ecological task practice, compensatory strategies, and group feedback. The group-based format may provide social support and may contribute to emotional regulation, perceived coping, and engagement, which are relevant factors in the experience of cancer-related cognitive impairment [23,24,38,39,40,41,42].

The difference in therapeutic contact and social support between G1 and G2 may introduce performance bias. However, this discrepancy is deliberate, as it enables an analysis of effectiveness and feasibility in a clinically realistic intervention format. The use of intention-to-treat analysis and linear mixed models will allow adjustment for individual variability and within-participant correlation over time. This comparison will help assess whether the multidimensional and group-based ALMA format is associated with greater improvement than individual cognitive training alone.

## 5. Clinical Integration and Future Directions

Should this study yield significant results, the findings have the potential to provide a replicable and empirically validated framework for implementation within clinical oncology practice. The efficacy of the aforementioned protocols (CT and ALMA) would allow for their integration into standard patient care plans. These protocols offer personalized cognitive support strategies that not only improve neurological outcomes but also the overall quality of life for breast cancer across the care continuum.

In view of these findings, it is recommended that subsequent research be conducted, encompassing an extended duration of the ALMA (extending from six months to one year) and recurrent assessments occurring every three or four months. Furthermore, a cognitive assessment is to be conducted six months after completion of the programme to assess the persistence of the stimulation’s effects.

## 6. Limitations

Despite the rigorous design of this protocol, several limitations must be acknowledged:

Generalizability: The study is restricted to a specific sample size and conducted within a single institution. This may limit the generalizability of the findings to more diverse oncological populations or different clinical settings.

Sample Homogeneity: The potential presence of clinical comorbidities—whether pre-existing or treatment-induced (e.g., fatigue, systemic toxicity)—may influence individual responses to the intervention and the stability of cognitive function.

Long-term Sustainability: A primary limitation is the absence of longitudinal follow-up beyond the four-month intervention period. This precludes the evaluation of the long-term sustainability of the cognitive intervention effects. To address this, further investigation is recommended, incorporating follow-up evaluations at 6 months and 1 year post-intervention to assess the persistence of cognitive gains.

Primary Endpoint Constraints: Another methodological limitation involves the selection of the MoCA as the primary outcome measure. While the MoCA is a highly reliable and extensively validated global screening instrument across clinical trials, it may be subject to ceiling effects when applied to relatively young and high functioning breast cancer survivors. Consequently, it could lack the necessary sensitivity to detect very subtle, domain-specific neurocognitive fluctuations associated with CRCI, introducing a potential risk of Type II errors. To rigorously counteract this inherent structural vulnerability, our protocol adopts a multi-method framework that supplements the MoCA with high-sensitivity secondary measures. By systematically cross-referencing objective, everyday ecological performance through the PECC and patient reported quality of life outcomes via the FACT-Cog, the study design ensures a comprehensive, multi-angle evaluation capable of capturing discrete cognitive changes.

Differential Contact (Hawthorne Effect): There is an inherent disparity in the level of therapeutic contact between groups. While the ALMA (G2) involves intensive, direct interaction with researchers, the control group (G0) receives only informational support. This difference in professional engagement and social support may independently influence patient outcomes.

Non-blinded investigator: A notable methodological constraint of this protocol pertains to the absence of a blinded evaluator for the neuropsychological tests. This is due to the fact that the same professional responsible for the initial and final assessments is also the one who conducts the ALMA group interventions. This arrangement, motivated by resource optimization and the need to maintain a consistent therapeutic relationship with the participants, could introduce detection bias or researcher expectations bias in the administration of the post-intervention instruments. Nevertheless, a range of strategies will be implemented to mitigate this risk and safeguard the integrity of the findings. These include standardizing assessment instruments, implementing statistical analysis without the researcher’s knowledge, ensuring that participants are unaware of the hypothesis, and utilizing robust modeling (LMM).

Additionally, to prevent subconscious cueing or expectancy effects during post-intervention (T1) evaluations, a rigid behavioral protocol will be enforced. This includes the strict use of manualized verbatim scripts for all test administrations to ensure identical phrasing, the systematic maintenance of a neutral affective posture (suppressing non-verbal reinforcements such as nodding or smiling, and avoiding differential verbal praise), and the use of precise digital timers to govern all response windows objectively, thereby completely eliminating investigator discretion.

Attrition Risk: The nature of the population (patients in active oncological treatment) poses a risk of dropout due to medical complications, fluctuating health status, or scheduling conflicts. While retention strategies are in place, high attrition could impact the statistical power of the final analysis.

## 7. Ethics and Dissemination

The findings of this study will be disseminated through peer-reviewed scientific publications, conference presentations, and academic communications. Results may also be shared with participating clinical services and relevant healthcare professionals involved in oncology, psycho-oncology, neuropsychology, and rehabilitation. Participants will be able to request a lay summary of the general study results once the trial has been completed and the main analyses have been finalized. No individual participant will be identifiable in any publication or dissemination material.

This study will be conducted in accordance with the ethical principles for medical research involving human subjects outlined in the Declaration of Helsinki. The protocol received favorable approval from the Research Ethics Committee of the Salamanca Health Area on 11 March 2024 (Reference: PI 2023 12 1478-TD).

### 7.1. Post-Trial Care

As this is a low-risk, non-pharmacological intervention trial, no specific post-trial medical care is anticipated beyond usual oncological care. Participants will continue to receive standard clinical follow-up from their oncology team throughout and after the study. If clinically relevant emotional distress, fatigue, anxiety, or other difficulties are identified during the study, participants will be referred to the appropriate oncology, psycho-oncology, or clinical support services according to the standard care pathway of the participating institution.

### 7.2. Compensation

Participants will not receive financial compensation for taking part in the study. Participation is voluntary, and refusal to participate or withdrawal from the study will not affect access to standard oncological care.

### 7.3. Informed Consent and Safety

Written informed consent will be obtained from all participants before any study-related procedure is conducted. Participants will receive an information sheet describing the objectives of the study, study procedures, randomization process, potential risks and benefits, confidentiality measures, voluntary nature of participation, and right to withdraw at any time without consequences for their usual oncological care. The model informed consent form and participant information sheet will be available in Supplementary Material S3 and retained in the study documentation according to the requirements of the Research Ethics Committee.

### 7.4. Data Protection and Confidentiality

All participant information will be treated as confidential and processed in accordance with Regulation (EU) 2016/679, the General Data Protection Regulation, and Spanish Organic Law 3/2018 on the Protection of Personal Data and Guarantee of Digital Rights. Each participant will be assigned a unique alphanumeric study code, which will be used in all study documents and databases. Only authorized members of the research team will have access to study data, and no directly identifiable participant information will be included in publications, presentations, reports, or shared datasets.

Detailed procedures for data collection, storage, confidentiality, quality control, backup, and change management are described in the Data Management and Confidentiality section.

### 7.5. Authorship and Dissemination

Authorship of publications derived from this trial will follow the recommendations of the International Committee of Medical Journal Editors. Authorship will be based on substantial contributions to the conception or design of the study, acquisition, analysis, or interpretation of data, drafting or critical revision of the manuscript, approval of the final version, and accountability for the integrity of the work.

The results of the trial will be disseminated through peer-reviewed scientific publications, conference presentations, and academic communications, regardless of the direction or magnitude of the findings. Results may also be shared with participating clinical services and relevant healthcare professionals involved in oncology, psycho-oncology, neuropsychology, and rehabilitation.

Participants will be able to request a lay summary of the general study results once the trial has been completed and the main analyses have been finalized. No individual participant will be identifiable in any publication, presentation, report, or dissemination material.

### 7.6. Protocol Amendments

Any relevant amendment to the protocol will be documented in writing, including the date, version number, description of the change, and rationale. Substantial modifications affecting participant safety, eligibility criteria, intervention procedures, outcome measures, statistical analysis, or data protection procedures will be submitted to the Research Ethics Committee for review and approval before implementation. Where appropriate, the trial registry will also be updated to reflect the approved amendment.

## Figures and Tables

**Table 1:** Flow of the Initial Assessment and Baseline Screening Phase.

Protocol Stage	Objectives	Components & Tools
**Stage 1: Clinical Screening & Informed Consent**	Establish eligibility, provide psychoeducation, and obtain legal authorization.	• **Informed Consent:** Signed authorization. • **Sociodemographic:** Age, education, and employment status.
**Stage 1: Clinical Screening & Informed Consent**	Establish eligibility, provide psychoeducation, and obtain legal authorization.	• **Clinical Profile:** Oncology history and specific pharmacological treatment.
**Stage 2: Neurocognitive & Psychometric Profiling**	Characterize the cognitive baseline and affective or sleep-related symptoms.	• **Performance & Function: ECOG** and **Lawton & Brody** scales. • **Objective Cognition: PECC** battery and **MoCA**. • **Subjective Cognition: FACT-Cog** and **MFE**. • **Psychometric Scales: Hamilton-A** (Anxiety) and **PSQI** (Sleep).

Note. ECOG: Eastern Cooperative Oncology Group Performance Status; PECC: Test for the Assessment of Everyday Cognition; MoCA: Montreal Cognitive Assessment; FACT-Cog: Functional Assessment of Cancer Therapy–Cognition; MFE: Memory Failures of Everyday Life Questionnaire; Hamilton-A: Hamilton Anxiety Rating Scale; PSQI: Pittsburgh Sleep Quality Index.

**Table 2:** Summary of Study Eligibility Criteria.

Inclusion Criteria	Exclusion Criteria
Women aged ≥ 18 years.	Major Neurocognitive Disorder (DSM-5) or neurodegenerative history.
Histologically confirmed diagnosis of breast cancer, stages I–IV. Patients with metastases or recurrence will be considered.	Primary or metastatic Central Nervous System (CNS) tumors.
Currently receiving active therapy (Chemotherapy, Radiotherapy, or Hormone therapy).	Severe sensory, motor, or language impairment preventing completion of cognitive tasks or questionnaires.
Written informed consent provided before any study-related procedure.	Current participation in other cognitive intervention trials.
Literacy level adequate for neuropsychological assessment.	Active substance abuse or severe psychiatric disorders.

**Table 3:** Summary of the primary and secondary outcome measures, assessment time points, scoring direction, and interpretation of improvement from baseline (T0) to post-intervention (T1).

Outcome Domain	Instrument	Time Points	Scoring Direction	Interpretation of Improvement
Objective global cognitive performance	MoCA v8.3	T0, T1	Higher scores indicate better cognitive performance	Increase from T0 to T1
Perceived cognitive function and cognitive-related quality of life	FACT-Cog v3	T0, T1	Higher scores indicate better perceived cognitive functioning and fewer perceived cognitive problems	Increase from T0 to T1
Everyday cognition	PECC	T0, T1	Higher scores indicate better every day cognitive performance	Increase from T0 to T1
Functional autonomy	Lawton and Brody Scale	T0, T1	Higher scores indicate greater independence in instrumental activities of daily living	Increase from T0 to T1
Anxiety symptoms	Hamilton Anxiety Scale	T0, T1	Higher scores indicate greater anxiety severity	Decrease from T0 to T1
Sleep quality	Pittsburgh Sleep Quality Index	T0, T1	Higher scores indicate poorer sleep quality	Decrease from T0 to T1
Performance status	ECOG Performance Status	T0, T1	Higher scores indicate poorer functional performance status	Decrease from T0 to T1
Everyday memory failures	MFE Questionnaire	T0, T1	Higher scores indicate more frequent everyday memory failures	Decrease from T0 to T1

Note. T0, baseline assessment; T1, post-intervention assessment; MoCA, Montreal Cognitive Assessment; FACT-Cog, Functional Assessment of Cancer Therapy-Cognition; PECC, Test for the Assessment of Everyday Cognition; ECOG, Eastern Cooperative Oncology Group Performance Status; MFE, Everyday Life Memory Failures.

## Data Availability

De-identified participant-level data generated during the study may be made available from the corresponding author upon reasonable request after publication of the main trial results. Data sharing will be considered only for scientifically sound proposals and will be subject to participant consent, approval by the Principal Investigator, applicable ethical and legal requirements, and institutional data protection policies. A data use agreement may be required before access is granted. No directly identifiable participants’ information will be shared.

## Supplementary Materials

The following supporting information can be downloaded at: https://www.mdpi.com/article/10.3390/jcm15134876/s1, Supplementary material S1. General Participant Information Sheet and Informed Consent Form; Supplementary Material S2. SPIRIT Schedule of Enrolment, Interventions, and Assessments; Supplementary Material S3. Materials to be used in the ALMA intervention.

## Protocol Version

This manuscript describes version 1.0 of the ALMA trial protocol, dated June 2026. Any subsequent modifications to the protocol will be documented with a new version number, date, description of the amendment, rationale for the change, and approval status by the relevant Research Ethics Committee, where applicable.
