# Supplementary material for: Effectiveness of the ALMA Intervention on Cognitive Function in Women with Breast Cancer: Protocol for a Randomized Controlled Trial
Source: J Clin Med. 2026 Jun 23;15(13):4876. doi: 10.3390/jcm15134876 (PMC13361397; doi:10.3390/jcm15134876)
Supplement: Supplementary file 1 [file jcm-15-04876-s001.zip › Supplementary material S1.pdf]

Journal of Clinical Medicine

MDPI

Supplementary material 1. General Participant Information Sheet and Informed Consent Form

1. Invitation to participate

You are being invited to take part in a research study because you are a woman diagnosed with

breast cancer and are currently receiving oncological treatment.

Before deciding whether to participate, you should read this information carefully and ask any

questions you may have. Participation is voluntary.

2. Purpose of the study

Some women with breast cancer experience cognitive difficulties during or after treatment, such as problems with memory, attention, concentration, planning, or everyday functioning.

The purpose of this study is to evaluate whether the ALMA intervention, a group-based cognitive intervention, may help improve cognitive functioning in women with breast cancer undergoing active oncological treatment.

3. What will happen if I participate?

If you agree to participate:

1. You will sign this informed consent form.

2. The research team will confirm that you meet the study criteria.

3. You will complete a baseline assessment.

4. You will be randomly assigned to one of three study groups.

5. You will follow the procedures of the assigned group for approximately four months.

6. You will complete a final assessment after the intervention period.

The assessments may include cognitive tests and questionnaires about perceived cognitive

functioning, everyday cognition, autonomy, anxiety, sleep quality, performance status, and memory failures.

4. Randomization

This is a randomized study. This means that, after the baseline assessment, you will be assigned by

chance to one of three groups:

G0: Health education control group

G1: Individual cognitive training group

G2: ALMA group intervention

You cannot choose the group. Participation in the study does not guarantee that you will receive the ALMA group intervention.

5. Study groups

Participants in G0 will receive standardized health education leaflets. Participants in G1 will complete an individual paper-and-pencil cognitive training programme over four months.

Participants in G2 will attend the ALMA group intervention, with two 120-minute sessions per week over four months.

After randomization, you will receive a specific information sheet explaining the group to which you have been assigned.

6. Possible risks or discomforts

This is a low-risk, non-pharmacological study. However, you may experience tiredness, frustration during cognitive tasks, emotional discomfort when discussing cognitive difficulties, or scheduling inconvenience. You may pause or stop an assessment or activity at any time. If necessary, you may be referred to the appropriate clinical support service.

7. Possible benefits

You may or may not receive direct personal benefit. The study may help improve future cognitive support interventions for women with breast cancer.

8. Voluntary participation and withdrawal

Participation is voluntary. You may withdraw from the study at any time without giving a reason.

Refusing to participate or withdrawing will not affect your oncological treatment, usual medical care, or relationship with your healthcare team.

9. Confidentiality and data protection

Your personal information will be treated confidentially. Each participant will be assigned a study code,and this code will be used in study documents and databases.

The file linking your identity with your study code will be stored separately and securely. Only

authorized members of the research team will have access to study data.

All procedures will comply with Regulation (EU) 2016/679, the General Data Protection Regulation and Spanish Organic Law 3/2018 on Personal Data Protection and Guarantee of Digital Rights. No directly identifiable information will appear in publications, presentations, reports, or shared datasets.

10. Results and dissemination

The results may be published in scientific journals, presented at conferences, or shared with

healthcare professionals. You may request a lay summary of the general study results once the study has been completed.

11. Ethics approval

This study has been approved by the Research Ethics Committee of the University of Salamanca /Salamanca Health Area.

Informed Consent Form

Study title:

Effectiveness of the ALMA Intervention on Cognitive Function in Women with Breast Cancer:

Protocol for a Randomized Controlled Trial

Participant code: ______________________

Please initial each statement:

[ ] I have read and understood the participant information sheet.

[ ] I agree to participate in this study.

Participant name: ____________________________________

Participant signature: _________________________________

Date: ____ / ____ / ______

Researcher name: ____________________________________

Researcher signature: ________________________________

Date: ____ / ____ / ______

Supplementary Material 1.2 Consent Sheets

Group-Specific Information Sheet: G0 Health Education Control
Assigned group: G0 — Health Education Control Group

Participant code: ________________________

You have been randomly assigned to the health education control group.
Participants in this group will receive standardized health education leaflets with recommendations on healthy lifestyle habits and self-care. These may include information about nutrition, physical activity, sleep hygiene, cognitive engagement, social participation, hydration, smoking cessation, and avoidance of excessive alcohol consumption.
You will not receive structured individual or group cognitive training during the study period.
You will complete the same assessments as the other groups at baseline and after approximately four months.
This group is important because it allows the research team to compare health education with the other study interventions.

Participation remains voluntary, and you may withdraw at any time without affecting your usual oncological care.

[ ] I confirm that I have received and understood the group-specific information sheet.

Participant name: _______________________________________
Participant signature: ____________________________________
Date: ____ / ____ / ______
Researcher name: _______________________________________
Researcher signature: __________________________________
Date: ____ / ____ / ______

Group-Specific Information Sheet: G1 Individual Cognitive Training

Assigned group: G1 — Individual Cognitive Training Group

Participant code: ________________________

You have been randomly assigned to the individual cognitive training group.

Participants in this group will receive a paper-and-pencil cognitive training dossier. The programme includes 80 structured activities over four months, approximately five activities per week.

The activities are designed to train cognitive functions involved in everyday life, such as attention,

memory, language, reasoning, planning, problem solving, and everyday cognition.

The programme is individual and non-tailored, meaning that all participants in this group receive the same set of activities. The research team will monitor progress through remote and in-person

follow-up contacts.

Possible discomforts may include fatigue, frustration during tasks, or temporary anxiety related to

perceived cognitive difficulties. You may pause or contact the research team if you experience

difficulties.

Participation remains voluntary, and you may withdraw at any time without affecting your usual

oncological care.

[ ] I confirm that I have received and understood the group-specific information sheet.

Participant name: ______________________________________

Participant signature: __________________________________

Date: ____ / ____ / ______

Researcher name: ______________________________________

Researcher signature: _________________________________

Date: ____ / ____ / ______

Group-Specific Information Sheet: G2 ALMA Group Intervention

Assigned group: G2 — ALMA Group Intervention

Participant code: _______________________

You have been randomly assigned to the ALMA group intervention.

ALMA stands for Playful Attention and Active Memory. It is a group-based cognitive intervention

designed for women with breast cancer who may experience cognitive difficulties during treatment. The programme will be delivered in face-to-face group sessions at the Faculty of Psychology of the University of Salamanca. It will last approximately four months and will include two sessions per week.

Each session will last 120 minutes, with a total of 32 sessions.

Each session will include:

25 minutes: welcome and psychoeducation

70 minutes: targeted cognitive stimulation

25 minutes: review, feedback, and closure

The activities may involve memory, attention, language, executive functions, reasoning, everyday

cognition, ecological tasks, compensatory strategies, and group feedback.

ALMA sessions will be delivered to small groups of up to six participants. Attendance will be recorded to monitor adherence. The study protocol defines completion as attendance at least 27 of 32 sessions, although you remain free to withdraw at any time.

Because the intervention is delivered in a group, other participants may hear information that you

choose to share. All participants will be asked to respect confidentiality, but the research team cannot fully guarantee what other participants may disclose outside the group.

Possible discomforts may include fatigue, frustration during cognitive tasks, emotional discomfort

when discussing cognitive symptoms, or discomfort related to group participation. You may pause, rest, skip an activity, or leave the session if needed.

Participation remains voluntary, and you may withdraw at any time without affecting your usual

oncological care.

[ ] I confirm that I have received and understood the group-specific information sheet.

Participant name: ____

Participant signature: ____________________________________

Date: ____ / ____ / ______

Researcher name: ____

Researcher signature: ____________________________________

Date: ____ / ____ / ______
