# Supplementary material for: Effectiveness of the ALMA Intervention on Cognitive Function in Women with Breast Cancer: Protocol for a Randomized Controlled Trial
Source: J Clin Med. 2026 Jun 23;15(13):4876. doi: 10.3390/jcm15134876 (PMC13361397; doi:10.3390/jcm15134876)
Supplement: Supplementary file 1 [file jcm-15-04876-s001.zip › Supplementary material S2.pdf]

Journal of Clinical Medicine

MDPI

Supplementary Material 2. SPIRIT Schedule of Enrolment, Interventions, and Assessments.

Key: X = procedure performed; ↔ = continuous/throughout period; G0 = health education leaflet/control; G1 = individual cognitive training; G2 = ALMA group intervention; T0 = baseline; T1 = post-intervention/close-out. Timepoints may be adapted to the journal template if required.

STUDY PERIOD	Enrolment	Allocation	Post-allocation				Close-out
**Study period / timepoint**	**Enrolment −t1**	**Allocation 0**	**Post-allocation Month 1**	**Post-allocation Month 2**	**Post-allocation Month 3**	**Post-allocation Month 4**	**Close-out T1**
**ENROLMENT**							
Eligibility screening	X						
Preliminary trial information	X						
Written informed consent	X						
Inclusion/exclusion criteria confirmation	X						
Sociodemographic data	X						
Clinical profile / oncology history / current treatment	X						
Participant code assignment / pseudonymization	X						
Baseline assessment (T0)	X						
**ALLOCATION**							
Stratification by baseline MoCA score		X					
Randomization, 1:1:1 allocation to G0/G1/G2		X					
Allocation concealment naintaid until completion of T0		X					

**INTERVENTIONS**							
G0: standardized health education leaflet / usual care		x					
G1: individual non-tailored cognitive training dossier		x	x	x	x	x	
G1: 5 cognitive training activities/week; 80 activities total			x	x	x	x	
G1: remote follow-up contact			x		x		
G1: in-person follow-up contact				x		x	
G2: ALMA group intervention, 2 sessions/week, 120 min/session			x	x	x	x	
G2: psychoeducation component			x	x	x	x	
G2: targeted cognitive stimulation component			x	x	x	x	
G2: group feedback / review component			x	x	x	x	
Intervention adherence monitoring / attendance log			↔	↔	↔	↔	
Retention strategies / reminders			↔	↔	↔	↔	
Withdrawal reasons documented if applicable			↔	↔	↔	↔	x
**ASSESSMENTS**							
Primary outcome: global cognitive performance — MoCA v8.3	x						x

Subjective cognitive function / cognitive QoL — FACT-Cog v3	X						X
Everyday cognition — PECC	X						X
Functional autonomy — Lawton & Brody Scale	X						X
Anxiety symptoms — Hamilton Anxiety Scale	X						X
Sleep quality — Pittsburgh Sleep Quality Index (PSQI)	X						X
Performance status — ECOG	X						X
Everyday memory failures — MFE Questionnaire	X						X
Adverse events / clinical changes			↔	↔	↔	↔	X
Concomitant oncological treatment update	X						X
Protocol deviations / intervention discontinuation			↔	↔	↔	↔	X
**DATA MANAGEMENT**							
Data entry in REDCap / database update	X		↔	↔	↔	↔	X
Data quality checks / missing data review							X
Preparation of pseudonymized dataset for statistical analysis							X

The schedule of enrolment, allocation, interventions, and assessments is presented following the SPIRIT participant timeline format.
