# Supplementary material for: Effectiveness of the ALMA Intervention on Cognitive Function in Women with Breast Cancer: Protocol for a Randomized Controlled Trial
Source: J Clin Med. 2026 Jun 23;15(13):4876. doi: 10.3390/jcm15134876 (PMC13361397; doi:10.3390/jcm15134876)
Supplement: Supplementary file 1 [file jcm-15-04876-s001.zip › Supplementary material S3.pdf]

Journal of Clinical Medicine

Supplementary Material 3. Materials to be used in the ALMA intervention.

Various topics related to chemobrain will be addressed as part of the psychoeducation programme. These topics will be presented by the researcher and discussed with the patients.

The topics will be:

- Neuroplasticity and its implications
- Mnemonic techniques and their applications
- The impact of cancer treatments on cognitive function
- Nutritional interventions for cognitive health
- Psychological aspects of body image and self-esteem
- The practice of mindfulness and its benefits
- Stress reduction techniques
- The benefits of physical exercise for cognitive function
- Assertive communication strategies
- Fear of cancer and its management
- Strategies for organizing tasks
- Training in "external aids"
- The impact of hormone treatment on cognitive function
- The question of whether chemobrain is a permanent condition Causes of cognitive decline.

➢ Attention

- Activities of similarities and differences, sequences of action.
- Search for keywords in texts.
- Double tasks (reading and counting), chained word activity.
- "Follow the sequence" game (palms, words, numbers), sequence colors.
- Learning music, games of synonyms and antonyms.
- Word search" (categories and letters), visual search tasks with increasing.
- Complexity (finding differences in detailed images).

  - Remember details from books or movies previously seen, solve complex, mental calculation problems.
  - Reverse Stroop Effect Activity.
  - Number of dictation with distractors.
  - Read texts and answer questions without looking back at them.
  - Reverse reconstruction: write down the previous day’s activities in reverse order.
- Memory
  - Visual word association (strategy: visualization).
  - Repeat the sequence of words (strategy: grouping), remember the story, sequence of words.
  - Categorize objects (strategy: acrostic).
  - -Pair game (strategy: spaced repetition), numerical and alphabetical sequence.
  - Shopping list, travel list, etc. (strategy: acrostics), visual memory game.
  - Sort objects by size (visual memory), spaced repetition.
  - Narrating and remembering personal stories, remembering a list of objects, remembering important dates.
  - Visual labyrinths (spatial memory), numerical processing.
  - Memory of recipes, sequence of recipes and remember objects, name objects with the assigned letter.
  - Advanced word sequence.
  - Cortex game.
  - Learning a list of related words and remembering them after a time interval, solving logical problems that require deductive reasoning.
  - Remembering long sequences of instructions and executing them in order.
  - Phonological fluency with restrictions: Examples: Say words that start with “P” but do not contain the letter “A,” words containing certain syllables, write words without certain vowels, etc.
- Calculation and logic activities
  - Mental math exercises.
  - Reasoning activities.
  - Riddles.
  - Math problems involving all arithmetic operations (easy, intermediate, and hard levels).

- Language
    - Creation of narratives is to be undertaken using a selection of words selected at random and subsequently narrated.
    - Detailed description is required of complex actions performed on a daily basis or hypothetical situations.
    - Creation of music.
    - Commit songs to memory and vocalize them.
    - Cumulative Story of Details
    - Fantastic Pair (Gianni Rodari): Choose two words that have nothing to do with each other (e.g., "dictator" and "butterfly") and write a micro-story that logically connects them.

- Executive Function
    - Sudoku puzzles.
    - Mazes.
    - Crossword puzzles.
    - Word search puzzles.
